# Supplementary material for: Chromothripsis during telomere crisis is independent of NHEJ, and consistent with a replicative origin
Source: Genome Res. 2019 May;29(5):737–49. doi: 10.1101/gr.240705.118 (PMC6499312; doi:10.1101/gr.240705.118)
Supplement: Supplemental Material [file supp_gr.240705.118_Supplemental_file_1.zip › contigs/annotated_contigs/DB113/contig.4.DB113_length_659_mean_cov_12.8012139605.docx]

**DB113_length_659_mean_cov_12.8012139605**

```
CTGTTAGAAATTGCTCCCCGGGGGCCTCCGGCATGAGAACCACTCGAACCTGGGTGTCAAAGCTCGCAGCGATCCAGATC|CACACAAC
                                                                                 >chrX:1
CGCACTCCAGCCTGACGACAGAGAGACTCCATCTCAAAAAAA|AAGAAAAGAAAAAAAGAAAGAAAGTGCT|CCCTTG|GGCCGGGCGC
43682321-143682373 + E=2e-01 p=2e-02        >chr9:130491079-130491107 + E=4e-02  >chr9
GGTGGCTCACGCCTGTAATCCCAGCACTTTGGGAGGCCGAGGTGGGCGGATCACGAGGTCAGGAGATCGAGACCATCCTGGCTAACACA
:136685550-136685746 - E=4e-85 p=0e+00
GTGAAACCCTGTCTCTACTAAAAATACAAAAAAAATTAGCCGGGCGTGGTGGTAGGTGCCTGTAGTCCCAGCTGCTTGGGAGGCTGAGG

CAGGAGAA|TGGCCAACATGGTGAAACCCTGCCTCTACTAAAAAATACAAAAAAATTAGCTGGGCGTGGTGGCACATGCCTGTAATCCC
          >chr10:12407859-12408158 + E=4e-168
AGCTACTCGGGAGGCTGAGGTGGGAGAATCCCTGGAACCTGGGAGCCGGAGGCTGCAGTGAGCAGAGATCGCACCAATGCACTCCAGTC

TGGGTGACAGAATGAGACTCCGTCTCAAAAAAACACGTTTCCTTCTCCTCAGTGTCAGTGTCACATTCACCTCTTGAAACATAGCTGTT

ATCTTTTGGGCAGGCTTCCTGTAGAAGTTGGGTCATATGTT
```
